# A Plea for More Robust Accountability Structures in the Global Strategy on Human Resources for Health: Workforce 2030

**DOI:** 10.3389/ijph.2023.1605603

**Published:** 2023-02-03

**Authors:** Oloruntoba Ogunfolaji, Hazem S. Ghaith, Olga Mbougo Djoutsop, Cynthia Kevine Wafo, Dorcas Phillipe Wafo, Ahmed Negida, Ulrick Sidney Kanmounye

**Affiliations:** ^1^ Research Department, Association of Future African Neurosurgeons (AFAN), Douala, Cameroon; ^2^ Faculty of Medicine, Université Technologique Bel Campus, Kinshasa, Democratic Republic of Congo; ^3^ Department of Anesthesiology and Critical Care Medicine, Faculty of Medicine and Biomedical Sciences, University of Yaounde I, Yaounde, Centre, Cameroon; ^4^ Department of Urology, Faculty of Medicine and Biomedical Sciences, University of Yaounde I, Yaounde, Centre, Cameroon

**Keywords:** global health, healthcare workforce, healthcare, human resources for health, African health

The Global Strategy on Human Resources for Health: Workforce 2030 [1] was adopted by the World Health Organization (WHO) member states in 2016. According to the Global Strategy, the world has a workforce deficit in excess of 18 million, and the majority of the deficit is in low- and middle-income countries (LMICs). [1] The Global Strategy proposed a multipronged approach to reducing the workforce deficit. This holistic strategy is informed by the labor markets, data and information systems, education and training, migration and mobility, and governance and planning. [1] The Global Strategy does a great job of developing targets and proposing health policy options to meet these targets. [1] It goes on to describe local and international stakeholders’ responsibilities and defines accountability structures. [1] For example, it details monitoring and evaluation plans in the short-, medium-, and long term.

Five years from this landmark event, it is critical that we assess our progress as a community and adjust the Global Strategy to address current challenges and seize opportunities. The COVID-19 pandemic represents the most important threat to meeting the 2030 goals. The World Health Organization estimates that more than 115,000 health workers have died from COVID-19. [2] COVID-related deaths of health workers will set back many countries, especially LMICs. The COVID-19 pandemic has also had a significant psychological burden on health workers in the form of burnout, depression, and post-traumatic stress. [3]

We must equally note that the pandemic offers opportunities for the Global Strategy. The pandemic highlighted weaknesses in our health systems, and our governments’ reluctance to strengthen health systems despite advice from leading public health agencies and experts did not go unnoticed. [4] The human resources for health community must seize this window of opportunity and remind stakeholders that human resources for health are an indispensable component of health systems. Also, we must remind them that investments in the health workforce will facilitate the attainment of multiple sustainable development goals. Finally, the human resources for health community must be part of ongoing demands for accountability regarding the management of COVID-19, as this will help strengthen governance and planning for future health system interventions.

Accountability is essential but difficult to implement, especially in global health. For example, Isaac Adewole, Nigerian Minister of Health from November 2015 to May 2019, represented Nigeria at the 69th World Health Assembly, where the Global Strategy was adopted. Yet, in 2018 he advised young Nigerian physicians to consider a career change in agriculture or tailoring. [5] The Democratic Republic of Congo faces multiple public health challenges, including malaria, HIV, the Ebola virus disease, cholera, and neglected tropical diseases. [6] These challenges are compounded by the lack of physicians, which is in part due to a low number of training spots. However, another cause is the defective pipeline between the students’ graduation and their availability in the labor market. First, the final year of medical school extends beyond 12 months and can be as long as 20 months in some cases. This phenomenon has been dubbed *année élastique* (elastic year) because the academic year stretches seemingly without end. [7] These elastic years are caused by delays between the different final year activities (i.e., neurology and psychiatry rotation, core rotations [medicine, obstetrics and gynecology, pediatrics, and surgery], viva evaluating the rotations, and research presentation). [7] After they graduate, the physicians cannot practice because they do not have their degree or license [7]. The waiting time for the acquisition of these two vital documents can be as long as 2 years. [7] As a result, these unlicensed physician graduates experience unemployment, contemplate migration, and change career focus.

The Global Strategy estimates Africa’s health worker needs-based shortage in 2030 at 6.1 million. [1] This deficit is the largest among the six regions and is expected to increase following the COVID-19 pandemic. [1, 2] We are confident that the Global Strategy can help solve the workforce deficit if it is implemented. However, we must set up better accountability structures. We look forward to reading articles on this topic in the *Global Strategy on Human Resources for Health: Workforce 2030*.
